# Clinical, Virologic, Immunologic Outcomes and Emerging HIV Drug Resistance Patterns in Children and Adolescents in Public ART Care in Zimbabwe

**DOI:** 10.1371/journal.pone.0144057

**Published:** 2015-12-14

**Authors:** A. T. Makadzange, M. Higgins-Biddle, B. Chimukangara, R. Birri, M. Gordon, T. Mahlanza, G. McHugh, J. H. van Dijk, M. Bwakura-Dangarembizi, T. Ndung’u, C. Masimirembwa, B. Phelps, A. Amzel, B. O. Ojikutu, B. D. Walker, C. E. Ndhlovu

**Affiliations:** 1 Ragon Institute of MGH, MIT and Harvard, Cambridge, Massachusetts, United States of America; 2 John Snow Inc, Boston, Massachusetts, United States of America; 3 Department of Medicine, University of Zimbabwe College of Health Sciences, Harare, Zimbabwe; 4 Department of Pediatrics, University of Zimbabwe College of Health Sciences, Harare, Zimbabwe; 5 HIV Pathogenesis Program, University of Kwa-Zulu Natal, Durban, South Africa; 6 African Institute of Biomedical Sciences, Harare, Zimbabwe; 7 United States Agency for International Development (USAID), Washington, DC, United States of America; University of Pittsburgh, UNITED STATES

## Abstract

**Objective:**

To determine immunologic, virologic outcomes and drug resistance among children and adolescents receiving care during routine programmatic implementation in a low-income country.

**Methods:**

A cross-sectional evaluation with collection of clinical and laboratory data for children (0-<10 years) and adolescents (10–19 years) attending a public ART program in Harare providing care for pediatric patients since 2004, was conducted. Longitudinal data for each participant was obtained from the clinic based medical record.

**Results:**

Data from 599 children and adolescents was evaluated. The participants presented to care with low CD4 cell count and CD4%, median baseline CD4% was lower in adolescents compared with children (11.0% vs. 15.0%, p<0.0001). The median age at ART initiation was 8.0 years (IQR 3.0, 12.0); median time on ART was 2.9 years (IQR 1.7, 4.5). On ART, median CD4% improved for all age groups but remained below 25%. Older age (≥ 5 years) at ART initiation was associated with severe stunting (HAZ <-2: 53.3% vs. 28.4%, p<0.0001). Virologic failure rate was 30.6% and associated with age at ART initiation. In children, nevirapine based ART regimen was associated with a 3-fold increased risk of failure (AOR: 3.5; 95% CI: 1.3, 9.1, p = 0.0180). Children (<10y) on ART for ≥4 years had higher failure rates than those on ART for <4 years (39.6% vs. 23.9%, p = 0.0239). In those initiating ART as adolescents, each additional year in age above 10 years at the time of ART initiation (AOR 0.4 95%CI: 0.1, 0.9, p = 0.0324), and each additional year on ART (AOR 0.4, 95%CI 0.2, 0.9, p = 0.0379) were associated with decreased risk of virologic failure. Drug resistance was evident in 67.6% of sequenced virus isolates.

**Conclusions:**

During routine programmatic implementation of HIV care for children and adolescents, delayed age at ART initiation has long-term implications on immunologic recovery, growth and virologic outcomes.

## Introduction

Over the last decade, access to antiretroviral therapy (ART) has substantially improved in low and middle income countries[1]. Significant improvements have been made in adult treatment while pediatric treatment coverage has lagged behind. Among adults, 64% of those needing ART are receiving it while only 34% of children in need of ART are on ART[1]. The demand for pediatric HIV and ART care has grown significantly over the years as treatment guidelines have shifted towards universal ART [2,3], and as children are surviving longer on ART. These children will need lifelong ART, with good long-term outcomes as they survive and mature into adolescents and adults living with chronic HIV infection[4].

Despite the treatment gap in pediatric HIV, increased access to ART for HIV infected children in sub-Saharan Africa has resulted in significant improvements in HIV related morbidity and mortality in children and adolescents[5]. As the number of children needing ART and surviving into adolescence has grown[6], the public sector in sub-Saharan Africa is playing an increasing role in providing HIV care. This is also occurring at a time when funding for HIV programs is flattening and governments in Africa are taking up an increasing share of the responsibility of providing HIV care services[7–9]. This funding transition is occurring at a time when outcome goals for HIV care are becoming increasingly more ambitious[10], i.e. 90% virologic suppression rates among individuals on ART. Data from routine implementation of pediatric and adolescent care within a low income country have been limited. To determine outcomes among children and adolescents in care within a public sector program, we analyzed clinical, immunological and virologic outcomes and the emergence of drug resistance among children and adolescents receiving care in one of the largest public ART programs in Zimbabwe.

## Methods

### Study Design

A cross-sectional evaluation of clinical outcomes, including virologic failure rates and patterns of emerging drug resistance among infants, children and adolescents (ages 0–19) on ART for at least 6 months at Parirenyatwa Hospital Family Care Center (PHFCC), was conducted between May-December 2012. Children had enrolled into care at the clinic between 2004–2011.

### Study Site

PHFCC, established in 2004, is a publicly funded, comprehensive HIV and AIDS Treatment and Care clinic in Harare, Zimbabwe. PHFCC is located within a public academic teaching institution where enrollment into care is open and referrals are not required. The Zimbabwe Ministry of Health and Child Care provides all resources including drugs, laboratory reagents, staff salaries and operational costs. HIV related medications such as antiretroviral therapy (ART), fluconazole, and cotrimoxazole are provided at no cost to the patient. Medical evaluations at the clinic are free of charge. A CD4+ T-cell count is routinely done at no cost to the patient. All other tests offered at PHFCC, including monitoring tests such as liver function tests, basic chemistries, chest X-rays are available, but must be paid for by the patient. At the time of this evaluation, resources for routine viral load monitoring were not available.

### Clinic Enrollment and Follow up Procedures

Patient eligibility for ART is based on national guidelines that are in concert with World Health Organization (WHO) guidelines. Children enrolled before 2008 were initiated on ART based on clinical staging or CD4+ T-cell counts[11,12]. Guidelines were further revised in 2008 recommending ART for all children <12 months regardless of CD4 count, and again in 2010 expanding the recommendation to include children 12–24 months of age[13]. All children received daily cotrimoxazole prophylaxis. Pre-ART counseling was provided to caregivers and with age-appropriate counseling to children and adolescents. Ongoing counseling, including adherence counseling, was provided at each clinic visit.

A paper file was generated for each patient at clinic enrollment. Socio-demographic characteristics, clinical history and physical examinations were conducted at the baseline enrollment visit and captured on a structured clinic form. Subsequent clinic visits that included anthropometric measurements, clinical evaluation and treatment plans were also noted in the medical record. The clinic lacks the resources or dedicated staff to identify individuals who have missed visits or to track patients that are lost to follow up.

### Study Procedures

We extracted data on demographic characteristics, caregiver history, clinical stage, tuberculosis history, basic anthropometric measurements, ART history, visit history, complete blood count, and CD4 T-cell profile from paper records and entered them into an electronic database. All data entries were entered in duplicate and verified.

To determine clinical and virologic outcomes, all children who presented to care during the study period and were on ART for at least 6 months were invited to participate in the study. A cross-sectional evaluation of consenting children and adolescents on ART at the clinic was conducted and included review of socio-demographic characteristics, clinical evaluation, CD4 count, viral load testing, and genotyping in those with evidence of virologic failure.

Genotypic drug resistance testing of samples from children with viral loads >1000 copies/ml was done. Viral sequences were performed in 102 viremic patients. Because of concerns for intermittent adherence to, or complete cessation of, therapy among adolescents with a possibility of reversion to wild type virus, we analyzed integrated viral DNA to identify both archived and ongoing HIV drug resistance mutations [14,15]. Previous data from Zimbabwe suggested good phenotypic concordance between plasma viral RNA and DNA analyses[16]. DNA was extracted from whole blood using a QIAamp DNA mini kit (Hilden, Germany). Nested PCR was used in amplification of the protease and reverse transcriptase genes. PCR products were purified using a QIAquick PCR purification kit (CA, USA). Sequencing reaction and purification were done using the Big Dye v3.1 (CA, USA) and a sodium acetate in-house method, respectively. Capillary electrophoresis was done on a 3130*xl* genetic analyzer using the Sanger dideoxy-sequencing technology. Sequences were assembled and edited to create consensus sequences using Sequencher v5.1 (Gene Codes Corp, MI, USA) software. Mutations were determined and analyzed using the Stanford University HIV drug resistance database (http://hivdb.stanford.edu/).

### Measures

The dependent variable was virologic failure, defined as a plasma viral load of >1000 copies/ml. Independent variables included demographic characteristics at cross-sectional evaluation (age at cross-sectional evaluation (years), gender, mother as caregiver, caregiver employment status, and disclosure of HIV status to the study participant), clinical characteristics pre-ART (age at ART initiation (years), time on ART (years), WHO stages 3 and 4 compared to stages 1 and 2, and history of pulmonary TB), ART regimen of nevirapine (NVP) compared to all other regimens, NNRTI ART regimen compared to protease inhibitor regimens, and severe immunosuppression on ART.

Severe immunocompromise was calculated based on the 2006 WHO treatment guidelines for age groups[12]: CD4 count<1500 cells/mm3 or CD4%<25% in children <12 months; CD4 count of <750 cells/mm3 or CD4% <20% in children ages 12–35 months, and CD4 count <350 cells/mm3 or CD4%<15% in children ≥60 months (5+ years). Height for age and weight for age z-scores were calculated using the WHO Anthro SAS macro v3.2.2[13].

### Data Analysis

Medians and interquartile ranges (IQR) were computed for continuous variables and counts with percentages for categorical variables. Study participants were categorized into four groups using pre-ART age as well as age at cross-sectional evaluation. Children were divided into two groups: infants and young children (<5 years), older children (5-<10 years). Adolescents were also divided into two groups: younger adolescents (10-<15 years) and older adolescents (15–19 years). Where there were insufficient numbers for smaller subgroup analyses, the cohort was divided into two groups: Children (<10 years) and adolescents (10+ years). Differences between children (ages <10 years) and adolescents (ages 10 to 19 years) were compared using chi-square statistics for categorical variables, and two sample t-tests comparing means for continuous variables. The non-parametric Wilcoxon rank sum test was used for CD4+ T cell count and percentage variables because the data were skewed. Paired data (CD4 percentage pre-ART and on ART) were compared using the Wilcoxon signed rank sum test.

Bivariate methods were used to examine the relationships of individual independent variables with the primary outcome of virologic failure, using one-predictor logistic regression models. Associations were estimated using odds ratios (OR) with 95 percent confidence intervals (CIs). Because predictors were associated with age, separate bivariate and multivariate models were created for two age groups based on age at ART initiation: children <10 years and adolescents 10 years and older. The multivariate logistic regression model included independent variables that were significant in the bivariate models defined as p<0.05, along with factors that are known to have an effect on virologic suppression. We tested for interactions among the independent variables in these models. The discrimination ability of the logistic models was measured by c-statistics with calibration assessed using Hosmer-Lemeshow chi-square statistics and their associated p-values. We employed an alpha of 0.05 in all statistical tests to determine statistical significance. All data management and statistical analyses were performed using SAS for Windows version 9.4 (SAS Institute, Inc., Cary, NC).

Written informed consent was obtained for all study participants. For children age 12 or younger, informed consent was obtained from his/her legal guardian; for children ages 13–17, consent was also obtained from his/her legal guardian and assent obtained from the child; and adolescents ages 18 and 19 were consented. The study was reviewed and approved by the local institutional review board of the Joint Research and Ethics Committee of the University of Zimbabwe, College of Health Sciences, Parirenyatwa Hospital.

## Results

### Demographic data

A total of 702 children and adolescents were enrolled in the cross-sectional evaluation. Table 1 shows the demographic characteristics of the 702 children and adolescents who were evaluated. Children were defined as those with age<10 years, and adolescents were defined as participants with ages 10–19 years. The median age of the cohort at the time of the cross sectional evaluation was 11.4 years (IQR 7.1, 16.0)(Table 1). There was no significant difference in gender distribution across the different age groups. Maternal caregiver status was assessed as a measure of maternal survival and involvement in the participant’s care. A significantly lower proportion of adolescents reported that their mother was their primary care giver as compared with younger children (34.4% vs. 68.3%, p<0.0001). Maternal HIV status was known for 461 children and adolescents (65.7% of the cohort). Among those whose mother was the primary care giver, 77.7% of mothers were HIV positive, 2.1% were HIV negative. The HIV status was unknown or not revealed in 20.2% and may reflect discomfort with disclosure to study staff in the presence of the child. Among those whose mothers were not the primary caregiver 49.6% were known to have been HIV positive, while the rest were of unknown HIV status. Primary caregivers were employed in 42.3% of the children and adolescents in the cohort.

**Table 1:** Demographic and baseline clinical characteristics of children and adolescents receiving HIV care in a public HIV program in Zimbabwe, by age at cross-sectional evaluation.

	Total		Infants & Younger Children		Older Children		Younger Adolescents		Older Adolescents		
			(<5 years)		(5 to <10 years)		(10 to <15 years)		(15 to 19 years)		
	N = 702		n = 103		n = 181		n = 210		n = 208		
	N	Median (IQR) or %	N	Median (IQR) or %	N	Median (IQR) or %	N	Median (IQR) or %	N	Median (IQR) or %	p-value^1^
**DEMOGRAPHIC**											
Current Age (years)	702	11.4 (7.1, 16.0)	103	3.6 (2.5, 4.2)	181	7.5 (6.2, 8.5)	210	12.4 (11.1, 13.5)	208	17.4 (16.5, 19.0)	
Gender (male)	341	48.90%	56	54.40%	86	47.80%	106	50.50%	93	44.90%	NS
Mother as primary caregiver	337	48.10%	74	71.80%	120	66.30%	84	40.00%	59	28.60%	<0.0001
Caregiver employed	279	42.30%	38	38.40%	76	44.70%	85	42.10%	80	42.60%	NS
**CLINICAL**											
Severe immunocompromise at baseline clinic enrollment^2^	384	59.40%	44	45.80%	103	58.90%	117	60.60%	120	65.90%	0.0221
Baseline CD4 cell count (cells/mm^3^)	608	358.5 (164.5, 696.0)	80	1039.5 (609.0, 1559.5)	160	538.5 (226.5, 851.5)	188	294.0 (155.0, 477.5)	180	231.5 (71.0, 384.0)	<0.0001
Baseline CD4 percent	530	13.0% (8.0, 20.0)	94	19.0% (12.0, 25.0)	160	14.0%(10.0, 20.0)	158	12.0% (7.0, 18.0)	118	9.9% (5.0, 16.0)	<0.0001
Baseline WHO clinical stage 1 & 2	186	26.50%	33	32.00%	48	26.50%	49	23.40%	56	26.90%	NS
Baseline WHO clinical stage 3 & 4	515	73.50%	70	68.00%	133	73.50%	160	76.60%	152	73.10%	
History of pulmonary TB at Baseline	257	37.80%	24	24.00%	55	31.40%	86	41.80%	92	46.20%	<0.0001

^1^Differences between children (<10 years) and adolescents (10 to 19 years) significant at p<0.05; NS, not significant

^2^ Severe immunocompromise was defined by aged group according to 2006 WHO treatment guidelines as CD4 count<1500 cells/mm3 or CD4%<25% in children <12 months; CD4 count of <750 cells/mm3 or CD4% <20% in children ages 12–35 months; CD4 count of <350 cells/mm3 or CD4% <15% in children ages 36–59 months; and CD4 count< 200 cells/mm3 or CD4%<15% in children ≥60 months (5+ years).

### Pre-ART Baseline Characteristics

Review of the clinic medical records provided baseline pre-ART data obtained at the time of enrollment into the HIV treatment and care program. Children in the cohort were enrolled with advanced clinical disease. Pre-ART clinic enrollment CD4+ T cell counts and CD4% were low (Table 1). Severe immunocompromise as defined by age specific absolute CD4+ T cell count and CD4% was present in 59.4% of participants and was higher in the adolescents as compared with the children (63.2% vs. 54.2% p = 0.0221). To allow for age specific changes in absolute CD4+ T cell counts, we focused our analysis on CD4% data. Median baseline CD4% in the cohort was low at 13.0% (IQR 8.0, 20.0), with the lowest median CD4% values observed among adolescents (Table 1). In combined analysis of children and adolescent patient groups, adolescents had significantly lower pre-ART baseline CD4% than children in the cohort (11.0% (IQR 6.0, 17.1) vs. 15.0% (IQR 10.0, 22.0), p<0.0001). Most of the children and adolescents were enrolled into care with baseline WHO clinical stages 3 & 4 (73.5%); the presence of advanced WHO clinical stage was similar in the children as in the adolescents (71.5% vs. 74.8%, p = NS). At baseline prior to ART initiation, 257 (37.8%) children and adolescents had a history of pulmonary tuberculosis (TB). A higher proportion of adolescents reported prior history of TB compared with children <10 years (44.0% vs. 28.7%, p<0.0001).

### Immunologic response on ART

The date of ART initiation was available from the medical record for 599 participants. To determine outcomes on ART and the impact of duration on ART on outcomes, we restricted our analysis to these 599 participants. The median age at ART initiation was 8.0 years and the median time on ART was 2.9 years (IQR 1.7, 4.5) (Table 2). The majority of the participants were on a nevirapine (NVP) based regimen (82.6%). Zidovudine (AZT) was used in the NRTI backbone in 21.8% of the cohort and stavudine (d4T) in 67.9% of the children and adolescents. The study was conducted prior to the widespread use of tenofovir (TDF) in first-line regimens in sub-Saharan Africa; 2.0% were on a TDF based regimen, with greater use of TDF among adolescents compared with children. Protease inhibitors were used during infancy (below age 3 years) and in the setting of treatment failure. All children and adolescents on a PI based regimen received Lopinavir/ritonavir.

**Table 2:** Immunologic and clinical outcomes on ART, overall and by age at ART initiation.

	Total		Infants & Younger Children		Older Children		Younger Adolescents		Older Adolescents		
			(<5 years)		(5 to <10 years)		(10 to <15 years)		(15 to 19 years)		
	N = 599		n = 201		n = 173		n = 168		n = 57		
	N	Median (IQR) or %	N	Median (IQR) or %	N	Median (IQR) or %	N	Median (IQR) or %	N	Median (IQR) or %	p-value^1^
Age at ART initiation (years)	599	8.0 (3.0, 12.0)	93	1.0 (1.0, 2.0)	153	4.0 (2.0, 6.0)	183	9.0 (7.0, 10.0)	170	14.0 (12.0, 15.0)	
Time on ART (years)	585	2.9 (1.7, 4.5)	198	2.8 (1.5, 4.5)	171	3.3 (1.8, 4.8)	164	3.1 (1.9, 4.6)	52	2.1 (1.2, 3.4)	NS
CD4 cell count (cells/mm^3^)	596	726.0 (426.0, 1099.5)	200	1108.5 (827.5, 1448.5)	173	801.0 (487.0, 1053.0)	167	164.0 (281.0, 726.0)	56	407.5 (269.5, 573.5)	<0.0001
CD4 percent^2^	496	25.0 (12.4, 38.1)	178	32.2 (19.9, 44.4)	132	28.1 (14.7, 38.3)	141	17.3 (5.9, 31.8)	45	11.2 (5.8, 21.7)	<0.0001
Severe Immunosuppression^3^	53	8.90%	5	2.50%	7	4.10%	32	19.20%	9	16.10%	<0.0001
Height for age z-score <-2 (height stunted)^4^	189	42.90%	48	30.60%	64	47.40%	68	53.50%	9	40.90%	0.0075
Weight for age z-score <-2 (underweight) for ages 10 years or less	33	14.40%	25	14.20%	8	15.10%		NA		NA	
BMI for age z-score <-2 (thinness)^5^	48	10.90%	14	8.90%	14	10.40%	17	13.40%	3	13.60%	NS
**TREATMENT**											
ART Regimen^6^											
D4T/3TC/NVP	334	61.30%	94	50.30%	99	66.40%	106	69.30%	35	62.50%	<0.0001
D4T/3TC/EFV	36	6.60%	3	1.60%	12	8.10%	13	8.50%	8	14.30%	
AZT/3TC/NVP	110	20.20%	64	34.20%	29	19.50%	14	9.20%	3	5.40%	
AZT/3TC/EFV	9	1.70%	2	1.10%	2	1.30%	5	3.20%	0	0.00%	
Protease inhibitor-based regimen	45	8.30%	24	12.80%	5	3.40%	10	6.50%	6	10.70%	
TDF regimen	11	2.00%	0	0.00%	2	1.30%	5	3.30%	4	7.10%	
HIV status disclosure	359	60.00%	29	14.50%	112	64.70%	163	97.00%	55	96.50%	<0.0001

^1^Differences between children (<10 years) and adolescents (10 to 19 years) significant at p<0.05; NA, data not available; NS, not significant

^2^CD4 percent was missing for 17.2% of patients.

^3^Severe immunodeficiency was defined by aged group according to 2006 WHO treatment guidelines as CD4 count<1500 cells/mm3 or CD4%<25% in children <12 months; CD4 count of <750 cells/mm3 or CD4% <20% in children ages 12–35 months; CD4 count of <350 cells/mm3 or CD4% <15% in children ages 36–59 months; and CD4 count< 200 cells/mm3 or CD4%<15% in children ≥60 months (5+ years).

^4^Height for age z-score was missing for 26.4% of patients.

^5^BMI for age z-score was missing for 26.4% of patients.

^6^ART regimen information was missing for 9.0% of patients. (D4T –Stavudine; 3TC–Lamivudine, NVP–Nevirapine, EFV–Efavirenz, AZT–Zidovudine, TDF–Tenofovir)

The proportion of children with severe age-defined immunocompromise decreased across all age groups on ART. PreART, 60.1% of the cohort had severe age-defined immunocompromise consistent with an immunologic diagnosis of AIDS, this dropped to 8.9% on ART (S1 Table). However, severe immunocompromise on ART remained significantly higher in adolescents compared with children. In children <10 years, the proportion with severe immunocompromise while on ART was less than 5%, compared with 19.2% and 16.1% in younger and older adolescents, respectively (Table 2).

**Fig 1:**
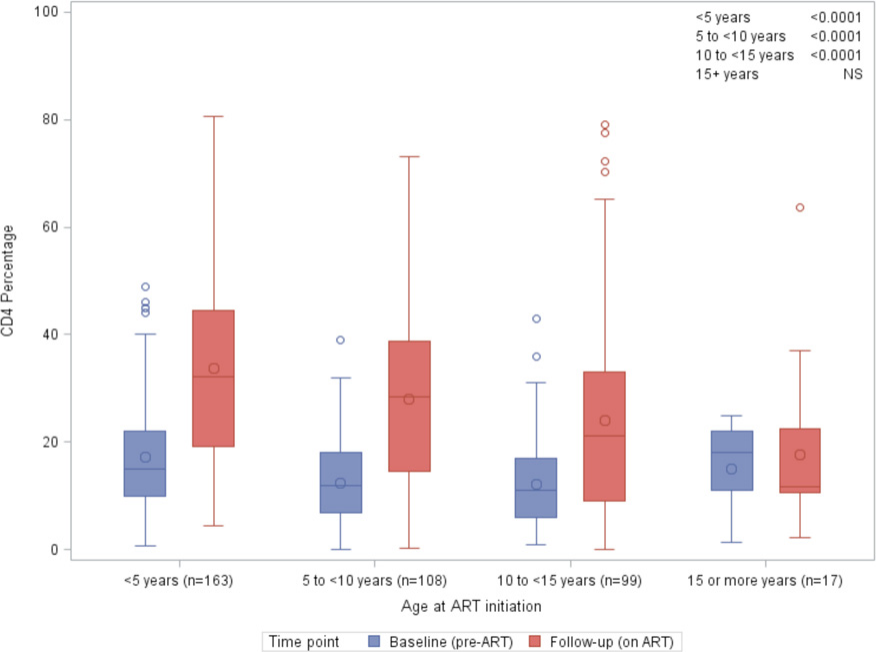
CD4 recovery on ART by age at ART initiation (n = 387).

The CD4% is considered to be a relatively stable measure of CD4+ T cell status that is less dependent on age than absolute CD4+ T cell count. There was significant recovery in absolute CD4+ T cell count and CD4% in all age groups (Fig 1A). The median CD4% on ART was lower in adolescents compared with children (14.8% vs 30.7%, p<0.0001) (Table 2). To evaluate immunologic recovery on ART, we compared pre-ART and on-ART CD4% in the different age groups based on age at ART initiation (Fig 1). CD4% was not routinely documented in the clinical medical record for children above the ages of 5 years, resulting in smaller numbers of older children and adolescents with pre-ART CD4%. A statistically significant increase (p<0.0001) between pre- and on-ART CD4% was observed in all age groups except for older adolescents (15–19 years) (Fig 1). The rise in median CD4% was 17.2% in children < 5 years, 16.5% in those ages 5-<10 years, and 10.2% in those ages 10-<15 years. The number of older adolescents with both a pre-ART and an on-ART follow up CD4% was low, (n = 17) and no significant change in CD4% was observed.

### Height and Weight

To determine the impact of ART and age at ART initiation on growth, we measured height and weight on ART and determined height-for-age and BMI scores. Stunting was higher among the adolescents when compared with children (51.7% vs. 38.4%, p<0.0075). The lowest rates of stunting were observed among children who initiated ART below the age of 5 years compared with those who initiated ART at older ages: 5-<10y (30.6% vs. 47.4%, p = 0.0032), 10-<15 years (30.6% vs. 53.5%, p<0.0001) (Table 2). There were no significant differences in the proportion of children with stunting among those who initiated ART at ages 15–19 years compared with those who initiated ART at age <5 years (40.9% vs. 30.6%, p = NS). This may suggest that some of the older adolescents may not be long term survivors of perinatal infection, and may have acquired infection more recently through sexual transmission. There were no significant differences in BMI measures across the different age groups.

### Virologic Failure

**Fig 2:**
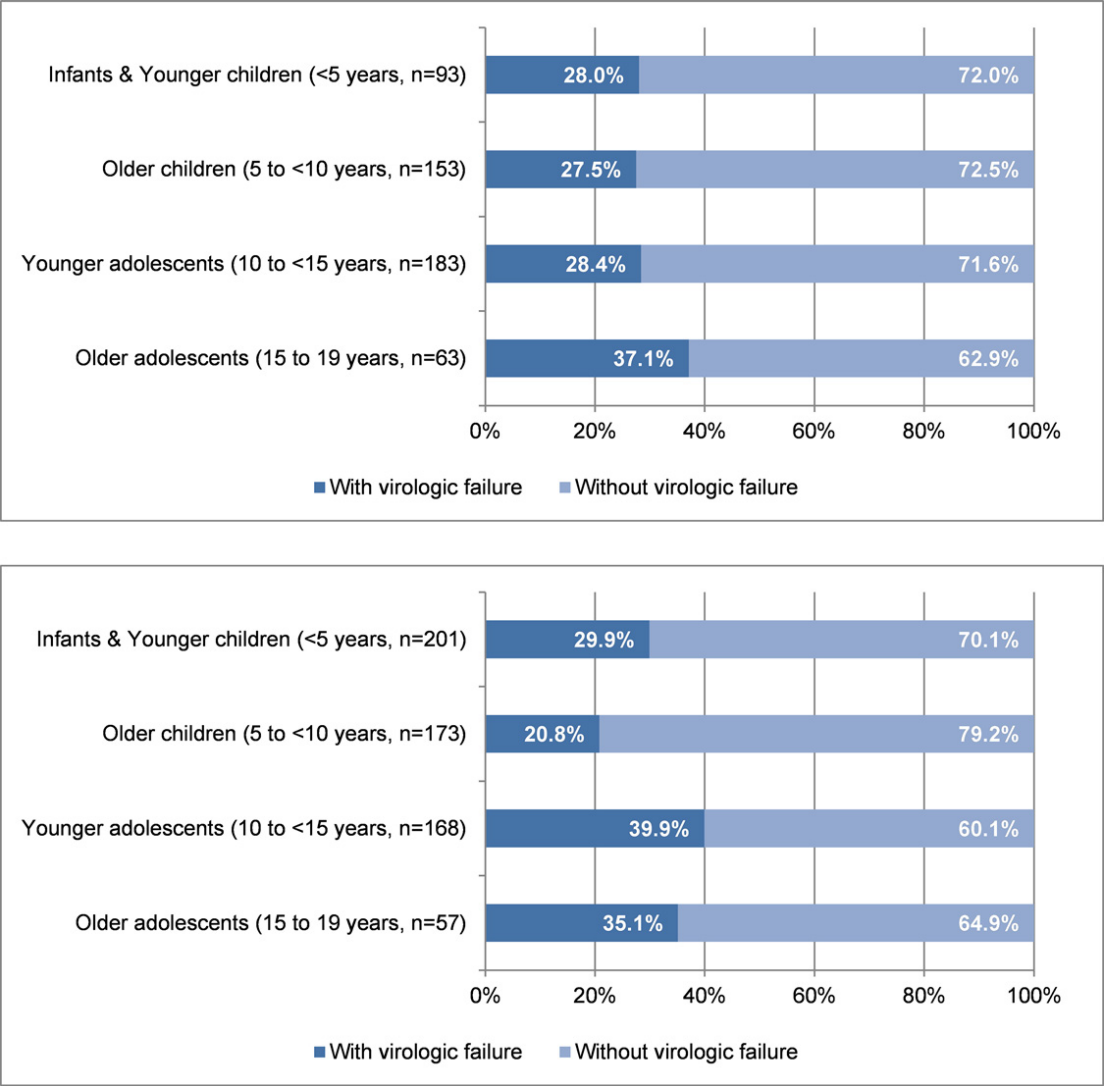
Virologic failure (a) by age group at cross-sectional evaluation (n = 599), (b) Virologic failure by age at ART initiation (n = 599).

We assessed virologic failure rates in the cohort and defined failure as an HIV VL>1000 copies/mL. On ART, 30.6% of study participants had evidence of virologic failure. Fig 2A shows virologic failure by age at cross sectional evaluation with further classification of pediatric and adolescent age groups. The prevalence of virologic failure was higher among older adolescents (ages 15–19 years), compared with other age groups, with 37.1% of the older adolescents having evidence of virologic failure (Fig 2A).

We evaluated the relationship between age at ART initiation and current virologic failure and found a significant association between them. The highest virologic failure rates were noted in those who initiated ART as adolescents (Fig 2B). Children who initiated ART between age 5 and 10 years had the lowest virologic failure rate (20.8%). In children who initiated ART below age 5 years, virologic failure rate was 29.9%. In combined group analysis, children who initiated ART below age of 10 years had lower virologic failure rates at the time of the cross sectional evaluation than those initiating ART in adolescence (ages 10–19 years) (25.6% vs. 38.6%, p = 0.0008).

Due to the significant impact of age at ART initiation on virologic outcome, we subsequently evaluated the factors associated with virologic failure based on age at ART initiation. In participants who initiated ART as children, age defined severe immunocompromise[12] was associated with a 3-fold increased risk of failure (AOR: 3.2; 95% CI: 0.9, 11.7, p = NS); however, this was not statistically significant (Table 3). A nevirapine based regimen was associated with virologic failure, with a 3 fold increased risk of failure among those on a nevirapine based regimen compared to those on a non-nevirapine based regimen (AOR: 3.3; 95% CI: 1.2, 8.7; p = 0.0180), adjusting for other factors. This was not observed among those who initiated ART as adolescents. In participants who initiated ART as children, there was no significant increased risk of failure with each incremental year on ART. However, when we grouped participants who initiated ART in childhood (<10 years) into those who had been on ART for less than 4 years or those on ART for 4 or more years, we found that virologic failure rates were higher among those who had been on ART for 4 or more years compared to those on ART for less than 4 years (39.6% vs. 23.9%, p = 0.0239).

**Table 3:** Factors associated with virologic failure.

	Virologic Failure			Virologic Failure		Virologic Failure			Virologic Failure	
	<10 years at ART initiation			<10 years at ART initiation		10+ years at ART initiation			10+ years at ART initiation	
				(N = 332)					(N = 214)	
	*Bivariate*			*Multivariate*		*Bivariate*			*Multivariate*	
	N	OR (95% CI)	p-value	AOR (95% CI)	p-value	N	OR (95% CI)	p-value	AOR (95% CI)	p-value
Age at cross-sectional evaluation (years)	374	1.0 (0.9, 1.0)	NS	0.9 (0.8, 1.0)	NS	225	1.0 (0.9, 1.1)	NS	2.4 (1.0, 5.9)	0.0486
Age at ART initiation (years)	374	0.9 (0.9, 1.0)	NS	- -	- -	225	0.9 (0.8, 1.1)	NS	0.4 (0.1,0.9)	0.0324
Male (ref: female)	373	1.3 (0.8, 2.1)	NS	- -	- -	225	0.8 (0.5, 1.4)	NS	- -	- -
WHO stage 3 & 4 (ref: stages 1 & 2)	374	0.8 (0.5, 1.3)	NS	- -	- -	225	0.9 (0.5, 1.7)	NS	- -	- -
Severe immunosuppression on ART (ref: not severe or no immunosuppression)	373	3.1 (1.0, 9.7)	NS	3.2 (0.9, 11.7)	NS	223	7.2 (3.3, 15.6)	<0.0001	8.4 (3.6, 19.6)	<0.0001
History of pulmonary TB (ref: no TB)	369	1.4 (0.8, 2.2)	NS	- -	- -	222	1.0 (0.6, 1.7)	NS	- -	- -
Mother as caregiver (ref: other relative)	374	0.9 (0.6, 1.5)	NS	- -	- -	224	1.1 (0.6, 2.0)	NS	- -	- -
Caregiver employed (ref: unemployed)	357	1.0 (0.6, 1.6)	NS	- -	- -	210	1.0 (0.6, 1.7)	NS	- -	- -
HIV status has been disclosed (ref: not disclosed)	373	1.0 (0.6, 1.6)	NS	- -	- -	225	3.9 (0.5, 33.0)	NS	- -	- -
NVP ART regimen (ref: other regimens)	336	3.5 (1.3, 9.1)	0.0109	3.3 (1.2, 8.7)	0.018	209	0.9 (0.5, 1.8)	NS	- -	- -
NNRTI ART regimen (ref: protease inhibitor regimens)	336	3.3 (1.0, 11.2)	NS	- -	- -	209	1.3 (0.4, 3.9)	NS	- -	- -
Time on ART (years)	369	1.1 (1.0, 1.2)	NS	1.1 (1.0, 1.2)	NS	216	1.0 (0.9, 1.2)	NS	0.4 (0.2, 0.9)	0.0379

OR, odds ratio; AOR, adjusted odds ratio; CI, confidence interval; NS, not significant

Among children who initiated ART as adolescents (10–19 years), for each additional year in age above age 10 years at the time of the cross-sectional evaluation, there was a 2-fold increased risk of failure (AOR: 2.4; 95% CI:1.0, 5.9, p = 0.0486), adjusting for other factors. In adolescents, severe immunocompromise was also associated with virologic failure. Those who started on ART as adolescents and had severe immunocompromise at the time of the cross sectional evaluation (CD4<200cells/mm^3^ or CD4%<15%) had an eight fold increased risk of virologic failure compared to those without evidence of immunosuppression (AOR: 8.4; 95% CI: 3.6, 19.6, p<0.0001). In participants who initiated ART as adolescents (ages 10 to 19 years), each incremental year in the age at which ART was initiated (AOR: 0.4; 95% CI: 0.1, 0.9, p = 0.0324), and each incremental year on ART (AOR: 0.4; 95% CI: 0.2, 0.9, p = 0.0379) were associated with decreasing risk of virologic failure.

#### Genotyping and resistance

To determine the prevalence of drug resistance in this cohort with high virologic failure rates, we analyzed drug resistance mutations circulating within this population. We anticipated that adherence may be intermittent, particularly in adolescents, and that circulating viral RNA may not accurately reflect the extent of drug resistance in the cohort, particularly in those who may have been evaluated at a time when they may have self-discontinued ART for a few weeks. We therefore analyzed proviral DNA for resistance. Analysis was restricted to those with data on age at ART initiation. A total of 102 sequential samples from participants with virologic failure underwent sequencing, representing 46.8% of those with evidence of virologic failure. We compared the cohort that underwent sequencing to those that did not, and found no significant differences (S3 Table) in all demographic, clinical and laboratory parameters analyzed including median viral load. There were no clinically significant mutations detected in 32.4% of sequences. Among those with at least one clinically significant mutation, a single mutation was present in 21.7% of sequences, and two or more mutations were present in 78.3% of sequences.

The most commonly identified mutation was the M184VI mutation that confers resistance to lamivudine (3TC), which occurred in 59.4% of those with at least one clinically significant mutation. The most commonly expressed NNRTI mutations in patients with resistance mutations were K103NRS (31.9%), and Y181CIF (37.7%). All of which decrease susceptibility to efavirenz and nevirapine which form the backbone of the national first line therapeutic regimen. E138AK is a common clade C polymorphism, occurred with high frequency and was detected in 24 isolates.

Thymadine Analogue Mutations (TAM1 and TAM2) mutations that confer resistance to NRTIs including Zidovudine and Stavudine were noted however at relatively low frequencies (13.0% and 21.7% respectively). Other NRTI mutations were relatively uncommon (Table 4). Protease inhibitor associated mutations and TDF-associated mutations (K65R) were infrequent, reflecting the small number of children and adolescents on those agents. There was no significant difference in the frequency of mutations by age at ART initiation.

**Table 4:** Resistance mutations by age group at cross-sectional evaluation.

	Total		Children (<10 years)		Adolescents (10–19 years)	
	(N = 69) ^1^		(n = 25)		(n = 44)	
Drug Resistance Mutation	Count	% of Isolates	Count	% of Isolates	Count	% of Isolates
**NRTIs**						
M184VI	41	59.40%	15	60.00%	26	59.10%
TAM1 (M41L, L210W, T215Y)	9	13.00%	3	12.00%	6	13.60%
TAM2 (D67N, K70R, 219Q)	15	21.70%	5	20.00%	10	22.70%
K65R	2	2.90%	0	0.00%	2	4.60%
L74IV	4	5.80%	1	4.00%	3	6.80%
Q151M	1	1.50%	1	4.00%	0	0.00%
**NNRTIs**						
K103N	22	31.90%	11	44.00%	11	25.00%
Y181C	26	37.70%	8	32.00%	18	40.90%
**PROTEASE INHIBITORS**						
L10F/I/R/V, V32I, M46I/L,	4	4.80%	2	8.00%	2	4.60%
I54V/M/L, V82A/F/T/S,						
I84V/A/C, L90M						

^1^ Isolates are from unique patients.

## Discussion

Increased access to ART for HIV infected children in sub-Saharan Africa has resulted in significant improvements in HIV related morbidity and mortality in children and adolescents[5]. However, these survival benefits are threatened as these children mature into adolescents, with increasing mortality rates among adolescents living with HIV infection[17]. Data from routine implementation of a national ART program in a largely public funded health care delivery system within a low income country have been limited and yet are important to guide policy and programmatic implementation.

Although prevention of mother to child transmission (PMTCT) programs have expanded in sub-Saharan Africa, early infant diagnosis remains poor[18,19], and a large number of children remain undiagnosed and present to care at older ages with advanced clinical disease. The data from this cohort shows that through 2012, the majority of children were enrolling into care and initiating ART outside of infancy and at older ages. The median age at ART initiation was 8 years, which is high compared with other described cohorts[20–22]. The majority of these children and adolescents are largely long-term survivors of perinatal infection with the sequalae of chronic infection such as advanced clinical disease (WHO stages 3 and 4), history of TB infection and severe immunologic compromise.

The children enrolling into care presented with low preART CD4% (CD4%<15%), a factor that is associated with increased risk of mortality[23]. In this cohort of children who have delayed ART initiation, we note poor overall immunologic recovery that persists even after several years on ART. In all age groups there was an improvement in CD4%; however children initiating ART at older ages had lower increases in CD4% on ART and median CD4% failed to rise above a threshold CD4% of 25% in adolescents. Young age, and high baseline CD4% were associated with better immunologic recovery, as has been described in other cohorts[24–28]. In adults, poor immunologic response is associated with increased risk of severe clinical events despite virologic suppression[29]. It is unclear if a similar effect is present in pediatric and adolescent populations. However, given improved survival, care providers for perinatally infected adults will need to monitor them for long term development of clinical events associated with poor immune recovery, particularly those who started ART at older ages.

We found a high rate of stunting among children and adolescents in this cohort. Stunting was associated with age at ART initiation. Children who initiated ART below age 5 years had lower rates of stunting than those who initiated ART above the age of 5 years. Comparative cohorts of HIV-infected children on ART in Europe and Africa note poor improvement in growth on ART among African children compared with European children, likely related to the delayed age at ART initiation[28,30,31]. Physically and socially disabling consequences of chronic HIV infection such as stunting and skin disease are often stigmatizing for infected children and adolescents[32,33]. Stunting in children is associated with poor long-term health and socio-economic outcomes[34], and is a common finding in chronic pediatric HIV infection. Improving early diagnosis, linkage to care for undiagnosed HIV infected children should raise preART CD4%s, improve long-term CD4+ T cell recovery and improve growth and developmental outcomes, particularly if ART is initiated in infancy.

We observed a high virologic failure rate in this cohort, raising concerns regarding long-term outcomes for adolescents and children with HIV in chronic care[20,21,35]. In the cross-sectional evaluation, and in all age groups, virologic failure was above 25%, peaking at 37% among older adolescents. This is far beneath current goals of achieving 90% virus suppression for those on ART[10]. Poor adherence is likely a primary driver of this finding, though other factors such as the pharmacokinetics and pharmacodynamics of ARVs in children with rapidly changing weight and metabolism should be considered. Adherence to ART in children and adolescents may be complicated by several factors, such as poverty, lack of caregiver involvement, inconsistent drug availability, and non-disclosure of HIV status[36–38], all of which contribute to the high virologic failure rates documented among children and adolescents in resource-limited settings[39–41]. In this cohort, in addition to identifying the high failure rates, we also note that among children and adolescents, unlike in adults, current age and age at ART initiation have an impact on virologic outcomes.

In children who initiated ART below age 10 years, nevirapine is an important driver of long term virologic outcomes. We found that nevirapine use was associated with a 3-fold increased risk of failure. This association was observed only among those who initiated ART in childhood and not those who initiated ART as adolescents. We also found higher virologic failure rates in children who initiated ART before age 5 years compared with those who initiated ART at ages 5–10 years. There were insufficient numbers in each age group to correlate this with resistance patterns observed. However, given the high prevalence of the Y181C and K103N mutations in the cohort, NNRTI resistance likely plays an important role in failure among children. This may be transmitted resistance or acquired resistance due to challenges associated with changes in drug metabolism with age, as well as weight-appropriate adjustments in dosing for children[42–44]. Although observational, our data raises questions on the use of nevirapine for first line treatment of perinatally infected children who present to care beyond infancy, and suggest consideration of PI or integrase inhibitor based regimens as the initial choices for children initiating ART below age 5 years. Current guidelines recommend the use of a PI based regimen for all children up to age 3 years[2]. We found no difference in outcome based on the NRTI backbone (i.e., stavudine compared with zidovudine), and found relatively low rates of TAM mutations among those with virologic failure.

Duration on therapy influenced virologic failure rates in children and adolescents on ART. In children initiating ART below the age of 10 years, continuous ART for 4 or more years was associated with increased virologic failure rates. The drivers of this are likely multifactorial and require further analysis, but may include inappropriate changes in dosing with the rapid changes in weight, height, body surface area or psychosocial factors such as treatment fatigue, and managing disclosure and adherence as the child grows and develops some independence over taking their own medications. This observation is in contrast to that observed among those who initiated ART as adolescents. Among those initiating ART as adolescents, incremental increases in age at ART initiation and duration on ART were associated with a decreased risk of failing on ART. We hypothesize that this may be due to increasing maturity, improving comprehension of the meaning of living with HIV, increased capacity to address complex emotions such as guilt, anger, stigmatization and an increased understanding of the consequences of non-adherence that comes with increasing age among adolescents. Further study of the biomedical and psychosocial drivers of improved virologic outcomes are needed. Improved understanding of the role that age at ART initiation, age at disclosure and route of HIV acquisition is needed to guide the development of appropriate psychosocial support mechanisms for children and adolescents living with chronic HIV infection.

We observed a high prevalence of clinically significant HIV mutations among those with virologic failure. The cross sectional nature of the laboratory component of the study, the absence of pre-treatment sequences or sequences from untreated children does not allow for conclusive determination that all mutations were ART associated and, indeed, some variants may reflect polymorphisms that may have increased in the presence of ART. However, most of the mutations identified were those associated with drug induced resistance[45]. The high frequency of resistance mutations and multiple mutations among individuals within in the cohort suggest that most children and adolescents are accumulating resistance mutations as they stay on failing regimens for prolonged periods, resulting in multi-class resistance. Implementation of routine affordable viral load monitoring will be necessary to ensure early detection of failure. Low cost genotyping is also urgently needed in routine care to identify the component of the cohort (32.4%) that merely requires strengthening of adherence. Premature regimen switches in these individuals are likely to have significant cost implications on national treatment programs.

The findings of this study are subject to some limitations. Data on nevirapine exposure for the prevention of mother to child transmission was inconsistently available, and some of the younger children in the cohort may have been exposed to nevirapine. Distinguishing between behaviorally infected adolescents and perinatally infected adolescents was also difficult in this cohort, as definitive information on timing and route of infection for most of these children was unknown. We anticipate that the vast majority were perinatally infected; however, sexually acquired infection cannot be excluded particularly among the older adolescents (15–19 years). Data on maternal characteristics such as HIV status, maternal treatment history were obtained, but deemed to be of low quality, as much of the data were recall by current caregivers.

The sample used has a survivor bias. Children in the cohort were those who survived and remained in care and consented to participate in the study. Data from those who did not present to care during the study period could not be obtained, and may potentially underestimate treatment failure rates. All the patients who presented during the study period were asked to enroll and to provide informed consent. We observed almost universal uptake into the study, as the study was nested within routine procedures and provided idealized care with viral load testing. Baseline data were obtained from patient medical records that were collected during routine clinical care in a very busy public non-research setting; as a consequence, there was some incomplete baseline information that could not be retrieved from patient files.

The data reflect care of adolescents in one of the largest public programs in Zimbabwe, and although it cannot be generalized to smaller community based practices, the results provide important insights into public care in a low-income country. During the period when the participants were enrolled, most pediatric and adolescent care was still mostly centralized. Increased efforts to decentralize care have occurred over the last 5 years and could potentially improve outcomes in this age group by reducing barriers such as transport costs, though this may be counterbalanced by limited clinical expertise among community based practices in the management of children and adolescents who may have complex clinical comorbid conditions and unique psychosocial issues.

In conclusion, children and adolescents in long term ART care, are at high risk of virologic failure and poor clinical outcomes due to late presentation to care and initiation of ART at older ages. Delayed ART initiation for perinatally infected children is associated with poor clinical outcomes including marked stunting and poor immunologic recovery. The 2015 updates to WHO guidelines have been expanded ART initiation criteria to allow for universal ART[3]. This should minimize delays in initiation among children and adolescents who link to ART care. However, delayed diagnosis remains a significant problem and improving access to early infant diagnosis and implementation of routine HIV testing of all children is necessary to facilitate early initiation of ART. High virologic failure rates are evident among children and adolescents in care. The mediators of virologic failure are likely multifactorial and we show that they are influenced by age. Virologic failure may be related to the therapeutic agents used such as Nevirapine, the pharmacodynamics and kinetics of drugs in children with a rapidly changing physiology, adherence and the neuro-psychosocial factors that influence it. Developing and increasing access to affordable, point of care technologies to facilitate therapeutic drug monitoring, improved viral load testing and HIV genotyping is necessary to guide dose adjustments, avoid prolonged virologic failure and prevent costly and unnecessary switches in therapy. This will need to be coupled with the development of evidence based psychosocial interventions to provide support to children and adolescents living with chronic HIV infection.

## Supporting Information

S1 Table Demographic and baseline clinical characteristics of children and adolescents receiving HIV care in a public HIV program in Zimbabwe, by age at ART initiation. (DOC)

S2 Table Immunologic and clinical outcomes on ART, overall and by age at cross-sectional evaluation. (DOC)

S3 Table Demographic and baseline clinical characteristics of children and adolescents with virologic failure, by resistance testing status. (DOC)
