# Supplementary material for: Clinical, Virologic, Immunologic Outcomes and Emerging HIV Drug Resistance Patterns in Children and Adolescents in Public ART Care in Zimbabwe
Source: PLoS One. 2015 Dec 14;10(12):e0144057. doi: 10.1371/journal.pone.0144057 (PMC4678607; doi:10.1371/journal.pone.0144057)
Supplement: S1 Table — (DOC) [file pone.0144057.s001.doc]

**S1 Table. Demographic and baseline clinical characteristics of children and adolescents receiving HIV care in a public HIV program in Zimbabwe**



**, by age at ART initiation**

|  | **Total** | | **Infants & Younger Children**  **(<5 years)** | | **Older Children**  **(5 to <10 years)** | | **Younger Adolescents**  **(10 to <15 years)** | | **Older Adolescents**  **(15 to 19 years)** | |  |
| --- | --- | --- | --- | --- | --- | --- | --- | --- | --- | --- | --- |
|  | N=599 | | n=201 | | n=173 | | n=168 | | n=57 | |  |
|  | N | Median (IQR) or % | N | Median (IQR) or % | N | Median (IQR) or % | N | Median (IQR) or % | N | Median (IQR) or % | p-value^1^ |
| **DEMOGRAPHIC** |  |  |  |  |  |  |  |  |  |  |  |
| Age at cross-sectional evaluation (years) | 599 | 11.2 (7.0, 15.8) | 201 | 5.4 (3.7, 7.2) | 173 | 11.0 (9.2, 12.4) | 168 | 16.0 (13.9, 17.4) | 57 | 19.1 (18.1, 19.8) | <0.0001 |
| Gender (male) | 277 | 46.3% | 103 | 51.5% | 76 | 43.9% | 71 | 42.3% | 27 | 47.4% | NS |
| Mother as primary caregiver | 289 | 48.3% | 140 | 69.7% | 82 | 47.4% | 55 | 32.9% | 12 | 21.1% | <0.0001 |
| Caregiver employed | 240 | 42.3% | 87 | 45.3% | 67 | 40.6% | 59 | 37.6% | 27 | 50.9% | NS |
| **CLINICAL** |  |  |  |  |  |  |  |  |  |  |  |
| Severe immunosuppression at baseline clinic enrollment^2^ | 338 | 60.1% | 109 | 56.2% | 105 | 64.8% | 101 | 65.2% | 23 | 45.1% | NS |
| Baseline CD4 cell count (cells/mm^3^) | 529 | 351.0 (164.0, 692.0) | 170 | 717.0 (349.0, 1161.0) | 156 | 298.0 (122.0, 572.0) | 152 | 235.0 (87.0, 405.5) | 51 | 292.0 (72.0, 442.0) | <0.0001 |
| Baseline CD4 percent | 468 | 13.0 (7.0, 20.0) | 185 | 14.0 (10.0, 22.0) | 139 | 12.0 (6.0, 18.0) | 121 | 11.0 (6.0, 17.0) | 23 | 15.9 (8.0, 22.0) | 0.0392 |
| Baseline WHO clinical stage 1 & 2 | 159 | 26.5% | 59 | 29.4% | 42 | 24.3% | 44 | 26.2% | 14 | 24.6% | NS |
| Baseline WHO clinical stage 3 & 4 | 440 | 73.5% | 142 | 70.6% | 131 | 75.7% | 124 | 73.8% | 43 | 75.4% |  |
| History of pulmonary TB at Baseline | 217 | 36.7% | 57 | 28.4% | 65 | 38.7% | 66 | 39.8% | 29 | 51.8% | 0.0175 |

^1^Differences between children (<10 years) and adolescents (10 to 19 years) significant at p<0.05; NS, not significant

^2^ Severe immunodeficiency was defined by aged group according to 2006 WHO treatment guidelines as CD4 count<1500 cells/mm3 or CD4%<25% in children <12 months; CD4 count of <750 cells/mm3 or CD4% <20% in children ages 12-35 months; CD4 count of <350 cells/mm3 or CD4% <15% in children ages 36-59 months; and CD4 count< 200 cells/mm3 or CD4%<15% in children ≥60 months (5+ years).
