# Supplementary material for: Clinical, Virologic, Immunologic Outcomes and Emerging HIV Drug Resistance Patterns in Children and Adolescents in Public ART Care in Zimbabwe
Source: PLoS One. 2015 Dec 14;10(12):e0144057. doi: 10.1371/journal.pone.0144057 (PMC4678607; doi:10.1371/journal.pone.0144057)
Supplement: S2 Table — (DOC) [file pone.0144057.s002.doc]

**S2 Table. Immunologic and clinical outcomes on ART, overall and by age at cross-sectional evaluation**

|  | **Total** | | **Infants & Younger Children**  **(<5 years)** | | **Older Children**  **(5 to <10 years)** | | **Younger Adolescents**  **(10 to <15 years)** | | **Older Adolescents**  **(15 to 19 years)** | |  |
| --- | --- | --- | --- | --- | --- | --- | --- | --- | --- | --- | --- |
|  | N=700 | | n=103 | | n=181 | | n=209 | | n=207 | |  |
|  | N | Median (IQR) or % | N | Median (IQR) or % | N | Median (IQR) or % | N | Median (IQR) or % | N | Median (IQR) or % | p-value^1^ |
| Age at ART initiation (years)^2^ | 599 | 8.0 (3.0, 12.0) | 93 | 1.0 (1.0, 2.0) | 153 | 4.0 (2.0, 6.0) | 183 | 9.0 (7.0, 10.0) | 170 | 14.0 (12.0, 15.0) |  |
| Time on ART (years)^3^ | 598 | 3.0 (1.7, 4.5) | 94 | 1.6 (1.0, 2.5) | 156 | 3.2 (1.7, 4.5) | 183 | 3.4 (1.8, 4.8) | 165 | 3.5 (2.5, 4.9) | <0.0001 |
| CD4 cell count | 698 | 726.0 (433.0, 1100.0) | 102 | 1278.0 (965.0, 1761.0) | 181 | 964.0 (654.0, 1227.0) | 209 | 724.0 (448.0, 932.0) | 206 | 409.5 (249.0, 624.0) | <0.0001 |
| CD4 percent | 581 | 23.8% (11.7, 37.6) | 101 | 28.8% (19.1, 40.0) | 142 | 33.8% (17.3, 44.4) | 164 | 26.0% (15.5, 37.6) | 174 | 11.4% (5.7, 26.2) | <0.0001 |
| Severe Immunosuppression^4^ | 62 | 8.9% | 6 | 5.9% | 4 | 2.2% | 11 | 5.3% | 41 | 19.9% | <0.0001 |
| Height for age z-score <-2 (height stunted)^5^ | 217 | 42.4% | 30 | 39.5% | 34 | 22.8% | 90 | 55.2% | 63 | 50.8% | <0.0001 |
| Weight for age z-score <-2 (underweight) for ages 10 years or less | 39 | 14.7% | 15 | 15.8% | 20 | 12.2% |  | NA |  | NA |  |
| BMI for age z-score <-2 (thinness)^6^ | 54 | 10.6% | 8 | 10.5% | 13 | 8.7% | 16 | 9.8% | 17 | 13.7% | NS |
| **TREATMENT** |  |  |  |  |  |  |  |  |  |  |  |
| ART Regimen^7^ |  |  |  |  |  |  |  |  |  |  |  |
| d4T/3TC/NVP | 388 | 61.2% | 37 | 36.6% | 92 | 58.2% | 117 | 65.4% | 140 | 72.2% | <0.0001 |
| d4T/3TC/EFV | 42 | 6.7% | 2 | 2.0% | 9 | 5.7% | 12 | 6.7% | 19 | 9.8% |  |
| AZT/3TC/NVP | 122 | 19.3% | 41 | 40.6% | 40 | 25.3% | 32 | 17.9% | 9 | 4.6% |  |
| AZT/3TC/EFV | 11 | 1.7% | 1 | 1.0% | 5 | 3.2% | 4 | 2.2% | 1 | 0.5% |  |
| Protease inhibitor-based regimen | 55 | 8.7% | 20 | 19.8% | 10 | 6.3% | 6 | 3.4% | 19 | 9.8% |  |
| TDF regimen | 16 | 2.5% | 0 | 0.0% | 2 | 1.3% | 8 | 4.5% | 6 | 3.1% |  |
| HIV Status Disclosure | 430 | 61.4% | 7 | 6.8% | 46 | 25.6% | 174 | 82.9% | 203 | 98.1% | <0.0001 |

^1^Differences between children (<10 years) and adolescents (10 to 19 years) significant at p<0.05; NA, data not available; NS, not significant

^2^Age at ART initiation was missing for 14.7% of patients.

^3^Time on ART information was missing for 14.8% of patients.

^4^ Severe immunodeficiency was defined by aged group according to 2006 WHO treatment guidelines as CD4 count<1500 cells/mm3 or CD4%<25% in children <12 months; CD4 count of <750 cells/mm3 or CD4% <20% in children ages 12-35 months; CD4 count of <350 cells/mm3 or CD4% <15% in children ages 36-59 months; and CD4 count< 200 cells/mm3 or CD4%<15% in children ≥60 months (5+ years).

^5^Height for age z-score was missing for 27.1% of patients.

^6^BMI for age z-score was missing for 27.1% of patients.

^7^ART regimen information was missing for 9.7% of patients. (D4T – Stavudine; 3TC – Lamivudine, NVP – Nevirapine, EFV – Efavirenz, AZT – Zidovudine, TDF – Tenofovir)
