# Supplementary material for: Clinical, Virologic, Immunologic Outcomes and Emerging HIV Drug Resistance Patterns in Children and Adolescents in Public ART Care in Zimbabwe
Source: PLoS One. 2015 Dec 14;10(12):e0144057. doi: 10.1371/journal.pone.0144057 (PMC4678607; doi:10.1371/journal.pone.0144057)
Supplement: S3 Table — (DOC) [file pone.0144057.s003.doc]

**S3 Table. Demographic and baseline clinical characteristics of children and adolescents**



**with virologic failure, by resistance testing status**

|  | **Children with  Resistance Testing** | | **Children without  Resistance Testing** | |  |
| --- | --- | --- | --- | --- | --- |
|  | n=102 | | n=116 | |  |
|  | N | Median (IQR) or % | N | Median (IQR) or % | p-value^1^ |
| **DEMOGRAPHIC** |  |  |  |  |  |
| Age at follow-up (years) | 102 | 13.3 (8.7, 16.5) | 116 | 13.6 (7.5, 17.0) | NS |
| Gender (male) | 52 | 51.0% | 52 | 45.2% | NS |
| Mother as primary caregiver | 51 | 50.0% | 47 | 41.2% | NS |
| Caregiver employed | 40 | 40.4% | 49 | 43.8% | NS |
| **CLINICAL** |  |  |  |  |  |
| Severe immunosuppression^2^ | 55 | 59.1% | 74 | 68.5% | NS |
| CD4 cell count (cells/mm^3^) | 90 | 305.5 (171.0, 661.0) | 106 | 334.5 (107.0, 620.0) | NS |
| CD4 percent | 73 | 14.0 (8.0, 18.0) | 89 | 10.0 (6.0, 16.6) | NS |
| WHO clinical stage 1 & 2 | 30 | 29.4% | 31 | 27.0% | NS |
| WHO clinical stage 3 & 4 | 72 | 70.6% | 84 | 73.0% |  |
| History of pulmonary TB | 35 | 35.7% | 51 | 44.7% | NS |
| HIV status has been disclosed | 77 | 75.5% | 73 | 63.5% | NS |
| **ON ART** |  |  |  |  |  |
| Age at ART initiation (years)^3^ | 86 | 9.0 (4.0, 12.0) | 97 | 8.0 (3.0, 13.0) | NS |
| Time on ART (years)^4^ | 87 | 2.9 (7.8, 1.9) | 95 | 3.3 (9.0, 1.5) | NS |
| CD4 cell count | 102 | 471.0 (254.0, 989.0) | 113 | 489.0 (272.0, 798.0) | NS |
| CD4 percent | 89 | 19.3 (6.7, 37.2) | 100 | 21.3 (8.8, 34.9) | NS |
| Severe Immunosuppression^2^ | 21 | 20.6% | 23 | 20.4% | NS |
| Height for age z-score <-2 (height stunted)^5^ | 39 | 51.3% | 38 | 41.2% | NS |
| Weight for age z-score <-2 (underweight)  for ages 10 years or less | 8 | 26.7% | 6 | 15.0% | NS |
| BMI for age z-score <-2 (thinness)^6^ | 5 | 6.6% | 9 | 10.2% | NS |
| **TREATMENT** |  |  |  |  |  |
| ART Regimen^7^ |  |  |  |  |  |
| d4T/3TC/NVP | 70 | 74.5% | 60 | 59.4% | NS |
| d4T/3TC/EFV | 3 | 3.2% | 9 | 8.9% |  |
| AZT/3TC/NVP | 14 | 14.9% | 17 | 16.8% |  |
| AZT/3TC/EFV | 1 | 1.1% | 4 | 4.0% |  |
| Protease inhibitor-based regimen | 5 | 5.3% | 7 | 6.9% |  |
| TDF regimen | 1 | 1.1% | 4 | 4.0% |  |
| Median viral load | 102 | 36850.0 (11200.0, 145000.0) | 116 | 40350.0 (10500.0, 241500.0) | NS |

^1^Differences between children with and children without resistance testing significant at p<0.05; NS, not significant

^2^ Severe immunodeficiency was defined by aged group according to 2006 WHO treatment guidelines as CD4 count<1500 cells/mm3 or CD4%<25% in children <12 months; CD4 count of <750 cells/mm3 or CD4% <20% in children ages 12-35 months; CD4 count of <350 cells/mm3 or CD4% <15% in children ages 36-59 months; and CD4 count< 200 cells/mm3 or CD4%<15% in children ≥60 months (5+ years).

^3^Age at ART initiation was missing for 16.1% of patients.

^4^Time on ART information was missing for 16.5% of patients.

^5^Height for age z-score was missing for 24.8% of patients.

^6^BMI for age z-score was missing for 24.8% of patients.

^7^ART regimen information was missing for 10.6% of patients.
